# A Medical Opinion

**Published:** 1878-05

**Authors:** 


A Medical Opinion.—The following is a true copy of a written opinion given by one who has an extensive practice: "Mr. ——, you have a lamnis of the Lung, turbit Lever, sower Stomak, and muskle affection of the Left Arm and right Hipp. I will help you conserable, but it is not a perfect cure for you."—*Toledo Med. Jour.*

